# Subchronic oral administration of crude khat extract (*Catha edulis* forsk) induces schizophernic-like symptoms in mice

**DOI:** 10.1186/s12906-016-1145-6

**Published:** 2016-05-31

**Authors:** Tegegne Bogale, Epherm Engidawork, Engida Yisma

**Affiliations:** Department of Pharmacy, Faculty of Medical and Health Sciences, Samara University, Samara, Ethiopia; Department of Pharmacology, School of Pharmacy, College of Health Sciences, Addis Ababa University, Addis Ababa, Ethiopia; Department of Nursing and Midwifery, School of Allied Health Sciences, College of Health Sciences, Addis Ababa University, Addis Ababa, Ethiopia

**Keywords:** Catha edulis forsk, Schizophernia, Mice, Crude khat extract, Subchronic administration

## Abstract

**Background:**

Chewing fresh leaves of the khat plant (*Catha edulis* forsk) is a deep rooted and widespread habit in East Africa and the Middle East. Although a body of knowledge exists about the adverse effects of khat on health, data are sparse with regard to the consequences of long-term khat chewing in resulting schizophrenic like symptoms.

**Methods:**

A crude extract of khat at different doses (100 mg/kg (K (khat)100), 200 mg/kg (K200) and 400 mg/kg (K400)) were administered for experimental group of mice whereas standard (ketamine (KT) 10 mg/kg- positive controls (KT10)) and vehicle (2 % v/v Tween-80 in distilled water – negative control groups (CON)) were administered for control groups of mice daily for two months to evaluate subchronic oral administration of crude khat extract to induce schizophrenic-like symptoms in mice. Mice were subjected to a battery of behavioural tests and parameters like locomotor activity, total time spent in social interaction and level of cognition among different groups of mice were measured and analyzed.

**Results:**

Khat at all doses significantly increased (*p* < 0.001) the mean locomotor activity score of mice compared to CON. However, the mean locomotor activity score of mice treated with khat was significantly lower (*p* < 0.001) compared to the mean locomotor activity score of KT10 mice (*p* < 0.001).

The mean total time score (in seconds) spent in social interaction, mean total time score (in seconds) spent in sniffing and following the partner was significantly higher (*p* < 0.001) in CON groups of mice compared to khat and ketamine treated groups. Moreover, in spatial memory task, the mean latency score (in seconds) to find the platform of khat and ketamine treated mice was significantly higher (*p* < 0.05) when compared to CON.

**Conclusions:**

Subchronic oral administration of khat showed an enhanced locomotor activity, reduced social interaction and impaired cognitive function, which demonstrated that long-term use of khat is associated with schizophernic-like symptoms.

## Background

According to World Health Organization (WHO) definition, schizophrenia is a severe mental disorder, characterized by profound disruptions in thinking, affecting language, perception, and the sense of self and it affects more than 21 million people worldwide [1]. Since no biological marker for schizophernia has yet been found, diagnosis is based on the assessment of the symptoms of each patient [2]. Different studies have shown that khat chewing is associated with occurrence of psychotic symptoms [3, 4].

Chewing the fresh leaves and shoots of the ever green plant khat (*Catha edulis*) dates back several centuries in eastern and southern Africa and the Arabian Peninsula [5]. Nowadays, it is estimated that 20 million people worldwide chew khat regularly to enjoy its psychostimulant effects [6].

Studies have shown that neurocognitive impairments [5], euphoria, excitability, anxiety, and insomnia [7, 8], are some of central nervous system effects of khat chewing. Khat chewing can also induce psychological dependency [5, 9, 10].

The main psycho-active component of khat leaves is cathinone (S-(−)alpha-aminopropiophenone) [11]. Cathinone resembles amphetamine in chemical structure and affects the central and peripheral nervous system [12, 13] and behavior [14, 15]. Similarly amphetamine and some of its derivatives have been shown to induce psychotic symptoms in experimental settings in humans [16, 17] and animals [18] and have been known to exacerbate psychotic states in psychiatric patients [19, 20].

To the best of our knowledge, the relationship between use of khat and psychiatric problems has been little explored in population-based studies. It has been postulated that khat use can exacerbate psychotic symptoms in individuals with pre-existing conditions, and precipitate psychiatric disorders in vulnerable subjects [14]. Moreover, khat consumption may affect the course of a psychotic disorder [16, 17].

Establishment of animal models of schizophrenia is critical for both understanding the mechanisms underlying severe mental disease and developing new antipsychotics [21]. There are various animal models of schizophrenia which seek to replicate the symptoms that are observed in human schizophrenics [21]. However, it has been difficult to find a particular model that is able to completely replicate all the symptoms observed in humans. In animal models, hyperactivity is considered as representative of positive symptoms in humans. Social withdrawal is considered to be the animal version of negative symptoms [21].

The current animal study was conducted in order to show interaction between khat use and schizophernia as there is no large-scale studies examining the relationship between effect of khat and schizophrenic-like symptoms. Therefore, the aim of this study was to evaluate schizophrenic-like symptoms of subchronic oral administration of crude khat extract in mice.

## Methods

### Drugs and chemicals

Diethyl ether and chloroform (Sigma-Aldrich, Germany), ketamine (Trittau, Germany) and 70 % ethanol (Pharmaceutical Fund Supply Agency (PFSA), Addis Ababa, Ethiopia) were purchased from the respective local vendors.

### Animals

Adult Albino mice bred in the animal house of the School of Pharmacy, Addis Ababa University and having weights ranging from 25 to 35 g and 8 weeks of age were used for the experiment. The animals were housed in groups of six in plastic wire meshed cages in the animal house. Wooden material used as bedding was replaced every 3 days. The animals were allowed free access to tap water and standard laboratory pellet. The environmental conditions were kept normal (25 ± 1 °C, and 12 h/12 h light/dark cycle). The study was approved by the Institutional Animal Care and Use Committee of School of Pharmacy, Addis Ababa University, Ethiopia and the care and handling of mice were done in accordance with the internationally accepted standard guidelines for use of animals [20].

### Plant material

Bundles of *Catha edulis* forsk shoots and small branches (about 8000 g) were purchased fresh at a local market in “Aweday”, one of the common natural habitat, which is located about 500 km away from Addis Ababa, in the Eastern part of Ethiopia. The bundles of khat plant were collected in December 2011 where large amounts of khat plant come to the local market. The fresh bundles were packed in plastic bags and transported in an ice box to the laboratory. The fresh leaves were immediately kept in a deep freezer (−20 °C). The plant was identified by a taxonomist and a voucher specimen was deposited in the National Herbarium, College of Natural Sciences, Addis Ababa University under a voucher Number 001.

### Preparation of plant extracts (crude extract)

The extract was prepared as described by Connor et al. [22]. The leaves were finely chopped in a dark place, weighed and placed in an Erlenmeyer flask containing reagent grade chloroform and diethyl ether in a 1: 3 v/v ratio. The volume of the combination of these volatile solvents used was more than that needed to just cover the minced leaves in the flask; hence a total of 400_ml solvents (100_ml of chloroform and 300_ml of diethyl ether) used for 200_g of the chopped leaves and shoots.

The mixture of khat plant with the solvent system was shaken using rotary shaker (120 rev/min) for two days under controlled temperature (20°c). The extract was decanted and filtered with Whatman No. 1 filter paper. The extract was placed in a hood under pressure for a week and then dried using Lyophilizer (Operan, Korea). Extracts were kept covered with aluminum foil and refrigerated.

### Induction of schizophernia

A study has shown that the most representative animal model of schizophrenic dysfunction seems to be those involving the administration of N-methyl-D-aspartic acid (NMDA) antagonist. Thus animals which were treated with NMDA antagonists e.g., ketamine could be used as a model of several aspects of schizophrenic dysfunction in humans [21]. Accordingly, Albino mice were injected intraperitoneally with 10 mg/kg ketamine for consecutive 14 days.

### Experimental design

A total of 60 albino mice were utilized and the animals were randomly divided into 5 groups of 12 animals (6 mice housed per cage) in each group.

- Group I- Normal mice (received the vehicle, 2 % v/v Tween-80 in distilled water, p.o [CON])
- Group II- Schizophernic control (received ketamine 10_mg/kg, i.p [KT10])
- Group III- Khat treated (received 100_mg/kg, p.o [K100])
- Group IV- Khat treated (received 200_mg/kg, p.o [K200])
- Group V- Khat treated (received 400_mg/kg, p.o [K400])

The extract was administered to the respective groups through oral route using intragastric tube (gavage) for 60 days. Such route was used since pharmacokinetics studies showed that *C. edulis* or S-(−)-cathinone is readily absorbed into plasma from the stomach and mouth [23, 24]. Besides, *C. edulis* leaves are usually taken orally in humans. Throughout the experimental period, all extract solutions were made fresh and containers including syringes were covered with aluminum foil to avoid light decomposition. On each day of the experiment, experimental mice were taken from their cage and weighed using electrical digital balance with 0.01 precision, since the weight of animals was necessary to determine the doses of khat extract.

Dose selection was made based up on a previous study done by Admassie et al. [25]. The dose of the extract required was determined based on the weight of the mice. Total weight of mice taking similar dose of extract was determined for ease of dose calculation. Once total amount of extract administered was calculated, the extract was reconstituted with 2 % v/v Tween-80 in distilled water. The volume of reconstitution fluid was adjusted, so that the maximum volume administered should not normally exceed 1 ml at a time for individual mouse in accordance with the Organization for Economic Co-operation and Development (OECD) guidelines for the testing of chemicals No. 413 ‘routes of exposure and dose administration considerations’: (as revised in 2010). CON group received only the reconstitution fluid based on their weight. This procedure was repeated every day for 60 days of experiment.

### Timeline of the study design

After mice were administered crude khat extract for 60 consecutive days, they were subjected to a battery of behavioural tests (locomotor activity study, social interaction study and cognitive function study). During all the behavioural studies, no extract treatment was given.

For each of the behavioral studies, 6 animals were utilized at a time out of 12 animals from their respective groups (CON, KT10 and khat treated). Hence, the same mice were utilized in different behavioural studies at different time.

### Locomotor activity study

The Linton instrumentation Activity Monitor (AM1053) (Palgrave, North folk), computer software coupled device, is used to record the activity score of mice. The device operates using an array of infrared beams and detectors.

Two activity boxes were placed in a quiet room. The boxes were transparent polycarbonate cages, which were similar to the home cages but were each equipped with 48 infrared beams, 24 on each level arranged in an 8x16, 1” (25.4_mm) pitched grid. A computer detected the disruption of the infrared beams and recorded the number of beam breaks. This beam breaks were considered as measures of locomotor activity.

Mice were habituated for two days to the testing room and the testing boxes (30 min/day for 2 days). Following habituation on day 1, mouse was placed into the locomotor activity boxes for 20 min and locomotor activity (number of infrared beam breaks) was measured. After each group study, the activity boxes were cleaned by 70 % ethanol to avoid any potential cues and make the activity box ready for the other group study. This procedure was repeated for another 4 days (a total of 5 testing days).

### Social interaction study

The apparatus used for the detection of changes in social behavior consists of an open-topped apparatus (51 × 51 cm and 20 cm high). Mouse used in this study was housed individually for 5 days. Mice were marked with different colours to distinguish the different groups and placed into the box (with 60 W bright illumination 17 cm above and video camera mounted above so as to view the entire apparatus).

One mouse from each group was taken at a time and social activity studies were done for each mouse individually over a 10- minute period by remote video recording. Once social activity study was done for a group, the open-topped box was cleaned with 70 % ethanol to avoid any potential cues and make the box ready for the other group study.

Total time spent in social interaction, total time spent sniffing and following a partner within an inch and half were also determined in the present study. Total time spent in social interaction was determined by timing the sniffing of partner, crawling under or climbing over the partner, genital investigation of partner and following the partner. Total time spent sniffing and following a partner were determined by timing the sniffing and following of the partner within an inch and half respectively.

### Cognitive study

Morris water maze (MWM) was used to evaluate cognitive function [26]. MWM consisted of a circular pool (122 cm diameter and 76 cm depth) in which mice were trained to escape from water by swimming to a hidden platform (1.5 cm beneath water surface) whose location could be only identified using distal extra-maze cues attached to the room walls. Different 3-D shapes that can act as visual cues were kept at constant location during the whole experiment. Water temperature was maintained at 21 ± 1 °C. The platform was placed in the middle of the North-West (NW) quadrant and remained at the same position during the whole experiment.

### Acquisition phase study

The spatial acquisition phase consisted of 16 training trials: 4 training trials per day and 4 training days with an inter-trial interval of 20 min. Mice were released randomly with their heads facing the pool wall from the four compass locations (NE, NW, SW, and SE), and allowed to swim and search for the platform for 120 s. If mice did not locate the platform after 120 s, animals were manually placed on the platform and allowed to remain on it for 30 s so that learning had to occur.

On the first training day, mice were given an acclimatization training session in the water maze by placing on the hidden platform and after acclimatization, mice were allowed to swim for 30 s and the latency to reach the hidden platform was recorded. And, if the mice were unable to found the hidden platform, they were guided back to the platform. This study was done for another 3 days (a total of 4 days) and the latency to reach the hidden platform in each day was recorded.

### Short-term memory retention phase study

One day after the acquisition phase (i.e., on day 5), subjects received a probe trial, in which the platform was removed. Mice were released from the NW start point and were allowed to swim freely for 60 s. Time spent in target quadrant (where the platform was located) was recorded.

### Long-term memory retention phase study

On the 12th day, subjects once again received the probe trial for 60 s to check retention of memory. Mice were released from the NW start point and were allowed to swim freely for 60 s. Time spent in target quadrant was recorded. Mice didn’t receive training between 5 and 12 days.

### Statistical analysis

Statistical analysis was performed using SPSS software package, Version 20.0. All the data were expressed as mean ± SEM. In order to study locomotor activity and social interaction among groups of mice, one way with Tukey’s procedure and repeated measures of ANOVA were utilized while for the study of cognition, one way repeated measure of ANOVA and paired sampled *t*-test was used. The value of *p* < 0.05 was considered to be statistically significant.

## Results

### Locomotor activity among groups of mice

**Fig. 1:**
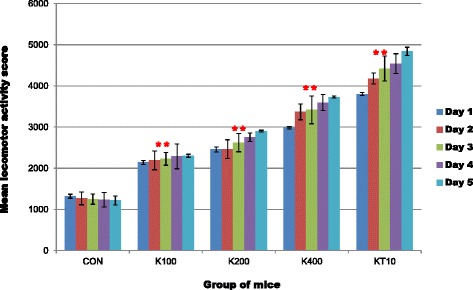
Mean locomotor activity score of groups of mice during the 20 min trial in the Activity Device Monitor across the five test days (*n* = 6/group). Data analysis were done by using Tukey’s procedure. **p < 0.001, indicates level of significance relative to controls (CON)

A one-way analysis of variance (ANOVA) was conducted to compare the locomotor activity scores of control, khat treated and ketamine groups of mice after subchronic crude khat extract administration daily for 2 months. ANOVA test indicated that there was a significant difference in the mean locomotor activity scores (across the five test days) of control groups (CON and KT10) and khat treated groups of mice (K100, K200 and K400) (F (4, 25) =136.17, *p* < 0.001) (Fig. 1).

A one-way repeated measures ANOVA was also conducted to compare the mean locomotor activity score of khat treated and ketamine groups of mice during the 20 min trial in the device across the five test days. There was a significant effect for days, Wilks’ Lambda = 0.58, F (4, 22) = 3.94, *p* = 0.015, multivariate partial squared = 0.42. This result suggest that the mean locomotor activity score of khat treated and ketamine groups of mice had significant effect for days.

Repeated measure of ANOVA also revealed that there was a significant effect between groups (CON and khat treated) of mice, F (4, 25) =135.82, *p* < 0.001. However, within-groups (days* group) effect of mean locomotor activity score was not significant, F (16, 100) = 1.25, *p* =0.246. This means that the mean locomotor activity score of each mice within respective groups of mice were not significantly different across the five test days.

### Social interaction

#### Total time spent in social interaction

A one-way analysis of variance was conducted to compare the total time spent in social interaction scores of control, khat and ketamine treated groups of mice after subchronic crude khat extract administration daily for 2 months.

**Fig. 2:**
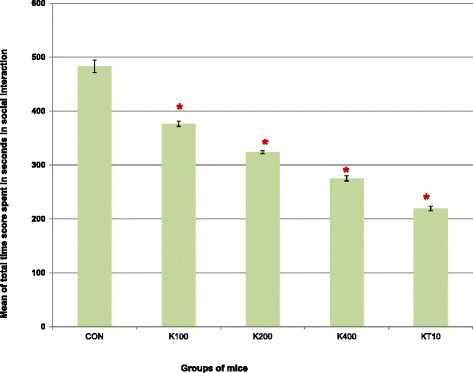
Mean total time score (in seconds) spent in social interaction among groups of mice over the 10 min video recording in open-topped box (*n* = 6/group). Pairwise comparisons revealed that the khat extract dose-dependently decreased the mean total time spent in social interaction (*p* < 0.001). *p < 0.001, indicates level of significance relative to controls (CON)

ANOVA test indicated that there was a significant difference in the mean total time spent in social interaction scores of control groups (CON and KT10) and khat treated groups of mice (K100, K200 and K400) (F (4, 25) =244.16, *p* < 0.001) (Fig. 2).

#### Total time spent sniffing the partner

**Fig. 3:**
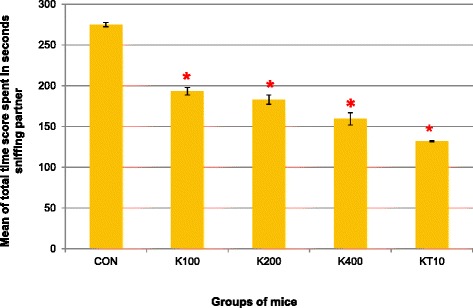
Mean total time score (in seconds) spent in sniffing partner within an inch and half of groups of mice over the 10 min video recording in open-topped box (*n* = 6/group). *p < 0.001; indicates level of significance relative to controls (CON)

A one-way analysis of variance was conducted to compare the total time spent in sniffing scores of control, khat treated and ketamine groups of mice after subchronic crude khat extract administration daily for 2 months. ANOVA test indicated that there was a significant difference in the mean total time score spent in sniffing partner within an inch and half of control groups (CON and KT10) and khat treated groups of mice (K100, K200 and K400) (F (4, 25) = 126.23, *p* < 0.001) (Fig. 3).

Pairwise comparisons of mean total time score (seconds) spent sniffing partner showed that when the different doses of crude extracts were compared to each other, the highest dose, K400, resulted in the lowest total mean time score spent in sniffing the partner compared to K200 (*p* = 0.014) and K100 doses (*p* < 0.001). However, pairwise comparisons among K200 and K100 doses were not significant (*p* = 0.57).

#### Total time spent following a partner

**Fig. 4:**
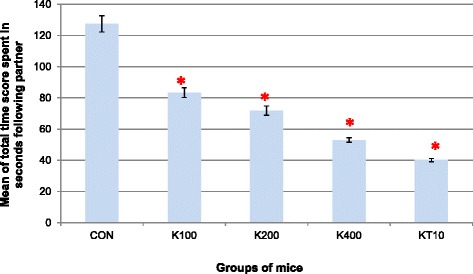
Mean total time score (in seconds) spent in following partner within an inch and half of groups of mice over the 10 min video recording in open-topped box (*n* = 6/ group). *p < 0.001, indicates level of significance relative to controls (CON)

A one-way analysis of variance was also conducted to compare the total time score spent in following a partner of control, khat treated and ketamine groups of mice after subchronic crude khat extract administration daily for 2 months. ANOVA test indicated that there was a significant difference in the mean total time score spent in following partner of control groups (CON and KT10) and khat treated groups of mice (K100, K200 and K400) (F (4, 25) = 115.60, *p* < 0.001) (Fig. 4).

Pairwise comparisons of mean total time score (seconds) spent following partner demonstrated that comparisons among different doses of the extract on the mean total time score spent following the partner revealed that the decrease was dose-dependent. Accordingly, K400 resulted in the lowest total mean time score spent in following the partner compared to K200 (*p* = 0.002) and K100 doses (*p* < 0.001). However, pairwise comparisons among K200 and K100 doses were not significant (*p* = 0.10).

### Cognition

The present results demonstrated the performance of khat treated and control groups of mice in water based spatial task. All groups of mice learned the task in the MWM within 16 trials. During the training most of khat as well as ketamine treated groups of mice preferred the edge of the wall and circled swimming in the direction submerged. In identification of the hidden platform, khat treated and ketamine groups of mice were able to touch the platform but unable to climb.

### Acquisition phase

**Fig. 5:**
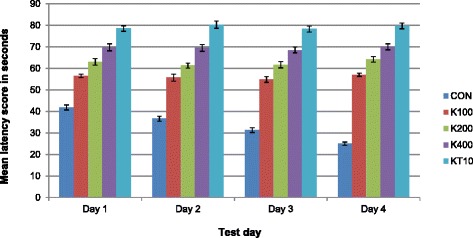
Mean latency score to reach the hidden platform (acquisition phase) among groups of mice over the period of four days of water maze trials (*n* = 6/ group)

The performance of acquisition phase was confirmed by applying the general linear model: repeated measures of ANOVA between the latency to reach the hidden platform and days of trials. The mean latency score of each group of mice during each test days are presented in Fig. 5. There was a significant effect for days, Wilks’ Lambda = 0.56, F (3, 23) = 6.14, *p* = 0.003, multivariate partial squared = 0.45. This result suggest that the mean latency score of khat treated and ketamine groups of mice had significant effect for days.

Repeated measure of ANOVA also revealed that there was a significant effect between groups (CON and khat treated) of mice, F (4, 25) =351.20, *p* < 0.001, multivariate partial squared = 0.98. Likewise, within-groups (days* group) effect of mean latency score to reach the hidden platform was significant, F (12, 75) =9.71, *p* < 0.001, multivariate partial squared = 0.61. This means that the mean latency to reach the hidden platform of each mice within respective groups of mice were significantly different across the five test days.

The mean difference between the latency score to reach the hidden platform on day1 and day 4 (∆L) was calculated for each khat treated and controls groups of mice in order to study their ability to learn the task. The means and SEM are presented in Table 1. One way ANOVA analysis indicated that there was a significant difference in the mean latency score to reach the hidden platform of CON and other groups of mice (K100, K200, K400 and KT10) (F (4, 25) = 26.73, *p* < 0.001). This indicated that CON group learns the task but other groups of mice failed to learn the task.

**Table 1:** The difference between mean latency score to reach the hidden platform on day 1 and day 4

Groups of mice	Mean ± SEM	N
CON	16.83 ± 1.08	6
K100	−1.17 ± 0.54	6
K200	−1.17 ± 2.0	6
K400	−0.17 ± 2.33	6
KT10	−1.0 ± 0.77	6

### Short-term memory retention phase

During the probe trial on day 5, time (in seconds) spent in the target and in the adjacent quadrants was analyzed using paired sample *t*-test and repeated measure of ANOVA. The result of the repeated measures of ANOVA indicated that there was a significant effect for quadrant (either target or adjacent quadrant) preferences, (Wilks’ Lambda = 0.03, F (4, 25) = 960.20, *p* < 0.001, multivariate partial squared = 0.98).

**Fig. 6:**
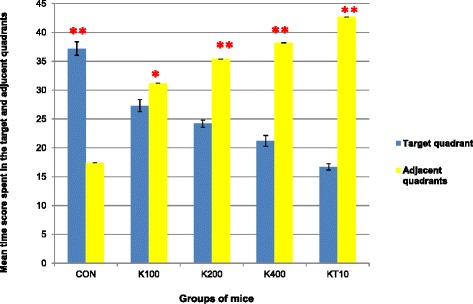
The mean time score spent in target and adjacent quadrants among different groups of mice during the probe trial on Day 5 (*n* = 6/ group). *p < 0.05, **p < 0.001; indicates level of significance for each group separately

The paired sample *t*-test (for each group separately) was also performed to confirm that the preference between the mean time score spent in the target and adjacent quadrants among different groups of mice. Accordingly, CON group of mice spent significantly more time in target quadrant (*p* < 0.001, t = 16.94) (Mean = 37.12, SEM = 1.17, *N* = 6) compared to adjacent quadrant (mean = 17.40, SEM = 0.00, *N* = 6). The increase was 19.77, with 95 % confidence interval between the means of 16.77 to 22.77 while K100 (t = 3.67, *p* = 0.014), K200 (t = 18.04, *p* < 0.001), K400 (t = 18.69, *p* < 0.001) and KT10 (t = 46.71, *p* < 0.001) groups of mice significantly spent more time in adjacent quadrant (See Fig. 6).

### Long-term memory retention phase

During the probe trial on day 12, time (in seconds) spent in the target and in the adjacent quadrants was analyzed using paired sample *t*-test and repeated measure of ANOVA. The result of the repeated measures of ANOVA indicated that there was a significant effect for quadrant (target and adjacent quadrants) preferences, (Wilks’ Lambda = 0.06, F (4, 25) = 366.04, *p* < 0.001, multivariate partial squared = 0.94).

**Fig. 7:**
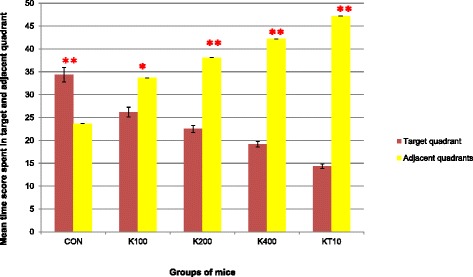
The mean time score spent in target and adjacent quadrants among different groups of mice during the probe trial on Day 12 (*n* = 6/ group). *p < 0.01, **p < 0.001; indicates level of significance for each group separately

The paired sample *t*-test (for each group separately) was also performed to confirm that the preference between the mean time score spent in the target and adjacent quadrants of different groups of mice. Accordingly, CON groups of mice spent significantly more time in target quadrant (*p* < 0.001, t = 6.73) (Mean = 34.33, SEM = 1.58, *N* = 6) compared to adjacent quadrant (mean = 23.67, SEM = 0.00, *N* = 6). The increase was 10.66, with 95 % confidence interval between the means of 6.59 to 14.74 while K100 (t = 6.95, *p* = 0.001), K200 (t = 20.43, *p* < 0.001), K400 (t = 38.33, *p* < 0.001) and KT10 (t = 66.48, *p* < 0.001) groups of mice significantly spent more time in adjacent quadrant (See Fig. 7).

## Discussion

This study described the schizophrenic-like symptoms of subchronic (two months) oral administration of crude khat extract (*Catha edulis* forsk) in mice using battery of behavioural tests (locomotor activity study, social interaction study and cognitive function study). The study revealed that subchronic oral administration of crude khat extract daily resulted in dose-related neurobehavioural abnormalities, including locomotor hyperactivity, abnormal social behavior and decreased cognition. These abnormal behaviors in mice are thought to be correlates of the symptoms of schizophrenia [27].

In this study, the mean locomotor activity score of mice treated with khat was significantly higher compared to control (*p* < 0.001) whereas it was significantly lower compared to ketamine treated groups of mice (*p* < 0.001). The tendency of increased locomotor activity with khat administration is concordant with previous studies [28, 29]. This phenomenon is assumed to be due to neurotransmitter systems, including dopamine and serotonin, as locomotor activities are modulated by these neurotransmitters. Cathinone, the active compound of khat is associated directly and/or indirectly with dopamine or serotonin release by its action on dopamine or serotonin transporter function [29, 30]. Further, it has been demonstrated that the motor activities induced by S – (−) cathinone in experimental animals are associated with dopamine release [29].

Habituation refers to a decrease in response that is observed when an identical stimulus is presented repeatedly and it is considered to be the simplest form of learning [31]. Interestingly, a deficit in this form of non-associative learning has been demonstrated in this study and the mean locomotory effect of control mice across the five test days decreased while this was not true for khat and ketamine treated mice. This might be probably because of control mice developed habituation of the device within test days. However, for those groups treated with khat and ketamine, the observed effect might be due to either neurochemical effect outweighs habituation of the activity device monitor or there was a deficit in non-associative learning.

Results from current study showed that subchronic oral administration of crude *C. edulis* extract induced the locomotor-activity in a dose-dependent manner. This finding is concordant with earlier study that demonstrated animals behave in the same way when administered cathinone as if they had been given (+)-amphetamine, and the response was dose related [32]. Animal study has also shown that repeated administration of amphetamine-like stimulants result in altered behavioural responses like behavioural sensitization [33]. Behavioural sensitization occurs when repeated treatment with psychostimulant drugs produces alteration in the behaviour that outlasts the initial neuropharmacological actions [33]. In this regard, khat extract in current study might exhibit behavioural sensitization on locomotion, ultimately leading to locomotor sensitization.

Psychosis and behavioural sensitization in laboratory animals are frequently correlated [34]. Therefore, in this study, the phenomenon of locomotor sensitization induced by *C. edulis* extract or ketamine in mice may bear striking similarities to the progressive development of psychosis in human. For example, different clinical studies revealed the association between repeated *C. edulis* chewing and psychosis [14, 15, 35]. Hence, the use of psychostimulants is often associated with a higher risk of psychosis [36, 37]. Taken together, the capacity of *C. edulis* to elicit a long-lasting behavioural sensitization supports the anecdotal reports about psychiatric problems which was associated with *C. edulis* chewing [15, 38]. Recently, it is also argued that organophosphates (through the exposure of khat to pesticides during or after cultivation) and high level of free radicals that could be linked directly to the effects of the khat constituents, cathinone and cathine, are important confounders in khat chronic consumption and psychosis symptoms in human [39].

It’s known that social withdrawal is considered to be the animal version of negative symptoms of schizophrenia [21]. The present study revealed the mean total time score spent in social interaction of khat and ketamine treated mice was significantly lower than control groups. Thus, khat treated groups were socially isolated than controls taking the vehicle but ketamine treated mice were highly isolated socially. This result is in line with the fact that schizophrenics experience symptoms subtracted from normal behaviour or function, for example social withdrawal, blunted affect and lack of motivation [21].

The current study also showed that after subchronic oral administration of crude *C. edulis* extract, the mean time (sec) spent in social interaction decreased in a dose dependent manner. This may be possibly explained because of khat-induced over stimulation of D2 receptors resulted in hypostimulation of D1 receptors which has been suggested to be in part responsible for negative symptoms of schizophrenia [40]. Furthermore, since the seminal work of Pycock et al.[41], many laboratories result have described reciprocal and opposite regulations between D2 and D1 systems. Hence, higher khat-induced over stimulation of D2 receptors because of the higher dose of crude khat extract administered (400 mg/kg) in current study may resulted in greater hypostimulation of D1 receptors.

According to Kwon et al., unfamiliar mice placed in a neutral arena will usually display high levels of sniffing and following [42]. In contrary, the results from this study indicated that time spent sniffing and following the partner within an inch and half showed decrement in a dose- dependent manner. This may possibly due to lack of motivation of mice taking the extract to investigate unfamiliar mice in the open box chamber indicating subtracted symptoms from normal behavior.

Since its first application in 1981, the MWM has become one of the most frequently used tool for analyzing spatial learning and memory [26]. The tool is known for its basic training protocols that include hidden-platform acquisition training, probe trial testing and working memory testing [26].

Our results indicate that there was mean improvement in overall performance of control mice in spatial learning whereas khat and ketamine treated mice showed very poor learning during the period of training. During the hidden-platform acquisition training, control mice learned the task with decreased latencies across time indicating mean improvement in the animal’s performance. The difference in latency to reach the platform between Day 1 and Day 4 was significantly higher in the controls indicating ability to learn the task. However, no significant learning was observed for either khat or ketamine treated mice during acquisition phase because of they showed no significant latency differences in the trials. This finding is consistent with a study that examined impaired cognitive function such as deficits in performance in spatial alternation tasks using ketamine in laboratory animals as compared to controls [43].

Previous preliminary observation of khat use suggests that chronic khat use is implicated with various cognitive and mental health impairments [18, 38]. However, extensive study on the effect of khat use on learning and memory is lacking. Therefore, the effect of khat use on learning and memory is usually deducted from studies made on other substances with close structural and functional similarity with the active constituent of khat. Amphetamine is the substance with close similarity to the active constituent of khat, cathinone [28]. A review by Warfa et al., indicated that chronic use of amphetamine leads to impairment on learning and memory, for instance, negative effect on episodic memory, working memory and a delay in the organizational and technical component of memory encoding [18].

In this study after a series of acquisition trial blocks, a probe or transfer trial at day 5 is usually performed to determine the spatial accuracy of the animal which is represented by the time it spends looking for the platform in the quadrant where the platform used to be (target quadrant) [44]. Accordingly, in the current study control group spent more time in target quadrant while K100, K200, K400 and KT10 groups spent more time in adjacent quadrants indicating that khat extract resulted in impairment of spatial accuracy to determine where the platform was used to be. The current study also indicated impairment in long term memory after probe trial at day 12 in MWM task. These all might be possibly resulted from the nature of cathinone. In CNS cathinone provokes the release of catecholamines especially dopamine at synaptic storage sites and results in overall stimulation of D2 receptors. This stimulation inhibits NMDA-mediated glutamate transmission and long term potentiation [45]. Also it is clear that glutamate and dopaminergic system are known to modulate each other’s activity in reciprocal fashion [46].

## Conclusions

Looking at the data produced in the present study, subchronic oral administration of crude khat extract resulted in enhanced motor activity in ADM study, symptoms subtracted from normal behavior of mice in open box chamber study and cognitive deficit in MWM study. Based on these findings, subchronic oral administration of crude khat extract (*Catha edulis* forsk) induces schizophernic-like symptoms in mice.

## Abbreviations

ADM, Activity device monitor; CNS, Central nervous system; MWM, Morris water maze; NMDA, N-methyl-D-aspartic acid; WHO, World Health Organization.
